# Hepatitis

**Published:** 1905-11

**Authors:** D. L. Field

**Affiliations:** Jeffersonville, Ind.


### HEPATITIS.

By D. L. FIELD, M.D., Jeffersonville, Ind.

There is one cause of inflammation of the liver which should not escape observation, and that is, the circulation of the blood through this organ is so slow, being computed to be only about one twenty-fifth part of the rapidity of other veins of the same size as those which distribute blood through the liver, without the aid of arterial action; thus resulting in such slow circulation, and therefore a choked, engorged state of the liver becomes a cause of inflammation. Nutrition and secretion of the various organs are always supplied from the arterial system, that of the bile alone excepted. It is sui-generis. To force the blood through the other parts there is exerted considerable *force*, but through the *liver* there is *none*, as it is expended, perhaps, more completely than in any other part of the body, by the length and minuteness of the ramifications of the blood-vessels in the abdomen, which are not equalled in any other of the whole system; hence the slowness of the current, and engorgement, favors liver disease. Languor, despondency, dull and heavy feeling accompany this state.

A rapid flow of blood is always necessary to health, but in the liver it is slow in order to secrete bile.

Hepatitis is acute and chronic, the acute being attended by pain, pyrexia, and nausea, frequent, strong and hard pulse, and high-colored urine. The chronic form is not characterized by the violent symptoms observed in the acute. There is dull pain in the right hypocondrium; a sense of fullness and heaviness; a dry cough, soreness and pain under the shoulder-blade, and great discomfort when lying on the right side.

Hepatitis is often communicated to the gall-bladder and biliary ducts, and is the cause of jaundice in such inflammations. The symptoms of dejection and debility, so conspicuous in *chronic* hepatitis, are the result of autointoxication from poisonous matters not eliminated by the liver.

It is asserted by some authors that the liver is, when unable to properly perform its function, more often the cause of melancholy, insanity, and suicide, than if the *brain* were diseased.

Why is toxamia and delirium, with low form of septic fever, akin to typhoid in its aspects, so common and fatal in hepatitis? With hepatitis we surely may expect duodenitis, inflammation and occlusion of the bile-ducts; and if so, the system becomes rapidly poisoned.

"In any case, the reality of the toxic nature of bile has long been believed in by the medical world, and has become experimentally established."

The daily secretion of bile is about 1,000 cubic centimeters, and during twenty-four hours a man makes, by the activity of his liver alone, an enormous quantity of poison. In eight hours a man forms enough to kill himself, simply by the hepatic secretion! In twenty-four hours the urine does not eliminate half the quantity of bile to poison a man; the urine of two days and a few hours would do so. Bile is nine times as poisonous as urine; in an equal period of time the bileary secretion represents a degree of toxic power six times as great as the urinary secretion! In hepatic disease it may be several days before the ecteric color appears. The urine is colored more quickly.

This much to prove how grave hepatitis is, and as prefatory to the few words I shall say on hepatitis.

To repeat, the symptoms of acute inflammation are rigors, fever, with pain of a dull character; fullness and tenderness over right hypochondrium; inability to lie on the right side; a dry cough, and if the deodenum is involved from contiguity—which I believe is the rule—there will be nausea and hiccough.

While the pain in the liver is dull, generally speaking, yet it is often a sharp, pricking pain, much like a pleuritic stitch. In the former the peritonial covering is supposed to be affected, while in the latter the whole viscus is congested and inflamed. The stomach disturbance is a marked feature of such diseases, and vomiting, with hiccough, is often so bad that radical measures directed toward the hepatitis are rendered inefficient. Till the hiccough and vomiting can be controlled very little satisfactory work can be done to allay the inflammation.

Rigors occurring and continuing during the disease should lead us to suspect a suppuration condition, particularly if there is hectic or septic fever and delirium.

I believe there is a chronic, congestive, torpid state of the liver, where the ducts become choked with a viscid secretion; in other words, a catarrhal condition, which eventuates in an inflammation from mechanical obstruction to the normal function of the organ. Such a state of the organ may go on for a long time before active phlogistic conditions supervene; and when they do develop we will find duodenitis present from occlusion of the bile-ducts and the absence of the normal aid and stimulus to it in disposing of the ingestu within it; therefore we have food accepted by the stomach to play havoc when it reaches the duodenum. Its presence there irritates, decomposes and poisons; in consequence we have with such hepatic trouble septic fever, rigors, sweats, incessant hiccough and low delirium. Such cases will die from auto-intoxication.

Acute hepatitis is almost sure to develop abscess, if the *gland itself* is inflamed; if the peritoneal surface, *no.* If the ducts and duodenum, gradual jaundice, septic fever and viliary toxemia.

The same causes which excite *acute* hepatitis will, in a less degree, cause *chronic* hepatitis.

The chronic liver trouble—which may at any time, from various causes, develop *acute* disease—is characterized, as was said before, by languor, lassitude, depression of spirits, hypochonriasis: sense of dread and an apprehension of impending evil. There is sallowness, muddy complexion, and usually gradual loss of flesh and strength.

It is doubtful if the medical profession has ever had any considerable knowledge of, or much success in, treating hepatic inflammation! I grant, though, in gall-bladder diseases, and heptic abscess, *surgial* science has accomplished brilliant results; but with all our mercury, salines, mineral water and counter-irritations, the disease goes on to a fatal termination. We may not cure *acute* hepatitis, but ought to be able to prevent the *chronic* form becoming acute!

Let us urge upon such liver-diseased persons an *active* instead of a sedentary life; abstinaance from *alcohol* and *gluttony;* walking instead of street-car, the automobile and the carriage; horseback riding; bathing and rubbing; the continuous use of mineral saline waters; and, above all, *keep the bowels open!*

